# Efficacy of two educational interventions about inhalation techniques in patients with chronic obstructive pulmonary disease (COPD). TECEPOC: study protocol for a partially randomized controlled trial (preference trial)

**DOI:** 10.1186/1745-6215-13-64

**Published:** 2012-05-21

**Authors:** Francisca Leiva-Fernández, José Leiva-Fernández, Fernando Zubeldia-Santoyo, Antonio García-Ruiz, Daniel Prados-Torres, Pilar Barnestein-Fonseca

**Affiliations:** 1 Family and Community Medicine Teaching Unit of Malaga, Distrito Sanitario Málaga, Málaga, Spain; 2 Vélez Sur Health Centre, Área Sanitaria Málaga Este-Axarquía, Vélez Málaga, Málaga, Spain; 3 Vélez Sur Health Centre, Área Sanitaria Málaga Este-Axarquía, Vélez Málaga, Málaga, Spain; 4 Pharrmacoeconomy and SRI Unit, Pharmacology and Clinical Therapeutic Department, Faculty of Medicine, Malaga University, Málaga, Spain; 5 Family and Community Medicine Teaching Unit of Malaga, Distrito Sanitario Málaga, Málaga, Spain; 6 Family and Community Medicine Teaching Unit of Malaga, Distrito Sanitario Málaga, Málaga, Spain

## Abstract

**Background:**

Drugs for inhalation are the cornerstone of therapy in obstructive lung disease. We have observed that up to 75 % of patients do not perform a correct inhalation technique. The inability of patients to correctly use their inhaler device may be a direct consequence of insufficient or poor inhaler technique instruction. The objective of this study is to test the efficacy of two educational interventions to improve the inhalation techniques in patients with Chronic Obstructive Pulmonary Disease (COPD).

**Methods:**

This study uses both a multicenter patients´ preference trial and a comprehensive cohort design with 495 COPD-diagnosed patients selected by a non-probabilistic method of sampling from seven Primary Care Centers. The participants will be divided into two groups and five arms. The two groups are: 1) the patients´ preference group with two arms and 2) the randomized group with three arms. In the preference group, the two arms correspond to the two educational interventions (Intervention A and Intervention B) designed for this study. In the randomized group the three arms comprise: intervention A, intervention B and a control arm. Intervention A is written information (a leaflet describing the correct inhalation techniques). Intervention B is written information about inhalation techniques plus training by an instructor. Every patient in each group will be visited six times during the year of the study at health care center.

**Discussion:**

Our hypothesis is that the application of two educational interventions in patients with COPD who are treated with inhaled therapy will increase the number of patients who perform a correct inhalation technique by at least 25 %. We will evaluate the effectiveness of these interventions on patient inhalation technique improvement, considering that it will be adequate and feasible within the context of clinical practice.

**Trial registration:**

Current Controlled Trials ISRTCTN15106246

## Background

Drugs for inhalation are the cornerstone of therapy in obstructive lung disease. Inhalers are the principle vehicles for the effective administration of medication. They allow high lung deposition of the drug and minimize systemic adverse drug reactions [1,2]. The effectiveness of drugs for inhalation can be influenced by many factors including age, sex, education of the patient, duration of disease, type of inhaler used, correct inhalation technique and use of several inhalers [2,3].

There are three main categories of inhalers: pressurized metered dose inhalers (pMDIs), dry powder inhalers (DPIs) and small volume nebulizers (SVNs). DPIs and pMDIs are the devices most commonly used for drug delivery in the treatment of asthma and COPD patients [1,2].

Technical features of inhaler devices have improved over time. However, the effectiveness in delivering drugs to the lung depends on correctly performed inhalation maneuvers. Incorrect usage of inhalers is a significant problem for both asthma and COPD management because it may result in diminished therapeutic effects, resulting in poor control of symptoms and thereby insufficient disease management [4-6]. As a result, patients might receive treatment, but without proper education and training in correct inhalation techniques, the therapeutic benefit is less than optimal [2].

In a previous study, we observed that up to 75 % of patients do not perform a correct inhalation technique (data not published) and other studies show a similar percentage of error [7-9]. Many inhalers are complicated to use and some require up to eight steps [1].

Efficient use of pMDIs requires coordination between simultaneous inhalation and device actuation, a slow and continuous inspiratory flow rate during inhalation followed by a breath hold of at least ten seconds. Patients frequently fail to exhale fully before inhalation, they activate the device before or at the end of inhalation, or while breath-holding. Other common errors are high inspiratory flows, not shaking the device before use and stopping inspiration when the spray collides with the throat. Other drawbacks of these devices are that they contain environmentally unfriendly propellants and most of them provide no dose counter, so the patients do not know the number of doses remaining and they use the device when it is empty [1,9,10].

The use of DPIs removes the coordination problem; however mistakes in the inhalation techniques are high. The most common errors are: not placing the device correctly, exhaling into the mouthpiece, low inspiratory flows and incorrect breath-holding. The most critical mistakes may be exhalation into the mouthpiece and low inspiratory flow, because the humidity and inadequate flow reduce the amount of drug released and its ability to reach the lungs [1,10].

Other factors associated with improper use of devices in patients with COPD are related to age and cognitive status [11,12], the number of devices [10,13] and the training of healthcare professionals. Healthcare personnel responsible for teaching the correct use of inhalation devices are lacking in basic knowledge and user skills [10,14]. To acquire the skills for using these devices, health professionals and patients must be adequately educated and trained [15,16].

The inability of patients to use their inhaler device correctly may be a direct consequence of insufficient or poor inhaler technique instruction. The quality of the initial instruction is of paramount importance for the outcome of inhalation therapy. Written instruction alone is insufficient in teaching correct inhalation techniques. Verbal instruction and technique assessment and reassessment are essential for patients to achieve a proper technique [17]. Patients who receive inhalation instructions at least once more after the initial instruction have better inhalation techniques compared with those who only receive instruction at the time of prescription [18]. Training in correct inhaler use rather than instructor demonstration appears to be important [19,20].

The objective of this study is to test the efficacy of two educational interventions to improve inhalation techniques in patients with COPD.

## Methods

This study has been approved by the Ethical Committees of Distrito Sanitario Málaga (01/03/07) and Axarquía (13/05/08).

### Participants

A total of 495 patients with COPD selected by a non-probabilistic consecutive sampling method from seven Primary Care Centers will participate in the study. The inclusion criteria will be: having been diagnosed with COPD by spirometry following the SEPAR (Sociedad Española de Neumología y Cirugia Torácica) guidelines [21], receiving clinical assistance in primary care centers in the Malaga area, having been prescribed inhalation treatment, and having agreed to be part of the study by giving signed written consent. Exclusion criteria will be: other respiratory conditions which are not included in the COPD definition (bronchiectasis, asthma or cystic fibrosis) and cognitive impairment problems registered in their clinical record (dementia, Alzheimer, Parkinson, cognitive decline). All these criteria will be reviewed in the patients´ clinical record.

### Sample size

This was calculated to detect a correct inhalation technique percentage difference between groups of 25 %, with a statistical power of 80 % and a confidence level of 95 %, assuming a percentage of expected losses of 40 %. The final sample size is 495 patients with COPD who meet the selection criteria mentioned above. In the Randomized group it will be necessary to include 297 patients, and in the Preferences group 198 (99 patients per arm).

### Design

**Figure 1:**
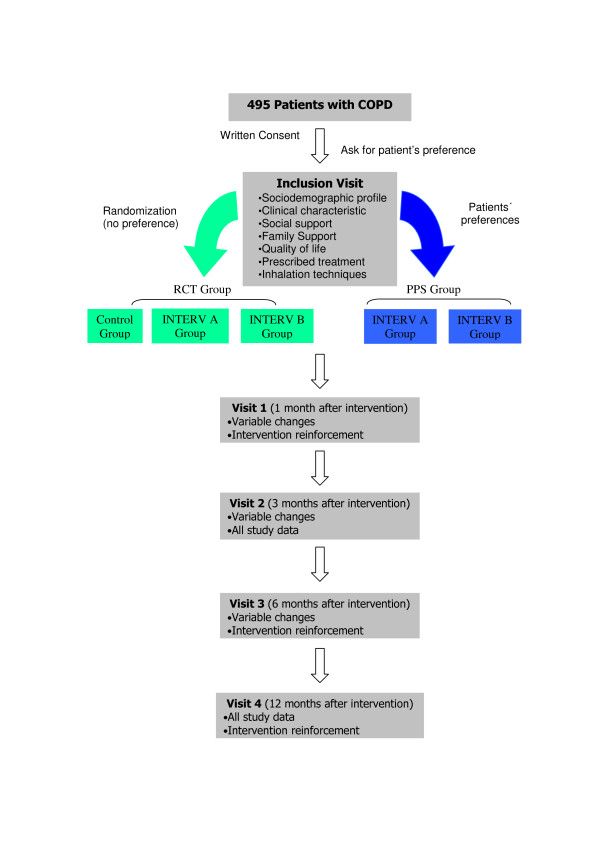
Study general graphic.

The study is structured as a multicenter patients´ preference trial or a comprehensive cohort design (Figure 1). The patients will be divided into two groups:

Randomized patients: randomized control trial (RCT) group with three arms (control, intervention A and intervention B)

Patients with preferences: PPS group with two arms (intervention A and intervention B)

Patients without strong preferences for a treatment are randomized and those with strong preferences are given their choice. Such a design enables comparisons between patients with and without a preference and an exploration of patient characteristics associated with preference [22,23].

### Setting

The study will take place in primary care facilities in Malaga, Spain.

### Outcomes

#### Primary outcome

Performance of correct inhalation techniques. The correct inhalation techniques will be measured by an investigator following SEPAR guidelines [1].

#### Secondary outcome

Participants will be evaluated using inhalatory pick flow, functional status (spirometry), mental-cognitive status (Mini-mental Status Examination test), dyspnea with Baseline Dyspnea Index (BDI) [24] and Modified Medical Research Council (MMRC) [25]; questionnaires of quality of life (St George respiratory questionnaire [26], and EuroQoL-5D [27]) and the clinical progress of illness (Seguimiento Clínico en EPOC: SeguiEPOC questionnaire).

#### Independent variables

Independent variables will be: age, sex, educational level, comorbidity, smoking history, COPD severity grade (according to SEPAR guidelines [28]), prescribed medication, family support (Family Apgar Test [29]): and social support (DUKE-UNC Test [30]).

### Intervention

We have designed two educational interventions:

Intervention A: written information.

We will give written information about inhalation techniques to the patient. We will design a leaflet about the correct inhalation techniques containing the main devices the patients use in our area. We will include four devices: Handihaler, Turbuhaler, Accuhaler and pMDI. We will describe all the steps necessary to perform the correct technique including images to illustrate the most relevant ones.

The patients included in this group will be asked how they use their inhalers and the interviewer will write down the mistakes in a template designed to follow the SEPAR guidelines [1]. When the inhalation techniques have been performed, the interviewer will give the leaflet to the patients and will invite them to read it and to identify differences between the steps of the correct inhalation technique (leaflet) and the technique they have performed.

In the follow-up visits we will ask the patients about the leaflet and about the differences they have found.

Intervention B: written information about inhalation techniques plus instructor training.

We will give written information about the inhalation technique to the patient (leaflet described above) and we will train the patient about correct inhalation techniques. The training of instructors in the use of inhaler devices has been carried out at the Pediatrics Neumology Service of Hospital Materno Infantil (Málaga). We will perform training in three steps:

Patients will be asked how they use their inhalers. Using a variety of placebo inhalers, all of them will demonstrate, to the instructor, how they use their inhalers.

When the patient has given the demonstration, the trainer will ask about the problems and perceived mistakes with the technique.

The trainer will demonstrate the proper technique. Each device will be used and its technique will be explained step by step. The importance of following the correct technique every time the patient uses the inhaler device will be emphasized.

Finally, the patients can ask questions and they will practice the techniques until they are correct or until the patient becomes tired.

In the follow-up visits we will review the inhalation technique and we will correct any mistakes or clear up any doubts as explained previously. The objective here is for the patients to identify their mistakes, and if they cannot, to remind them of the proper technique by giving as many demonstrations as necessary.

### Recruitment

Patients will be contacted using their health center records. They will be invited to participate in the study after a brief explanation by telephone about the research aims and they will receive an appointment in the health center. At this first appointment (inclusion visit) patients will receive more detailed information about the study and, if they agree to participate, they will sign the written consent.

At this point, subjects will be asked whether they have a preference for one of the interventions and they will be divided into two groups (Figure 1). Those who select the preference group choose their intervention and they will form two arms of the study. The participants without a preference will join the randomized group and they will be randomly allocated into the three arms of this group.

In the preferences group, the two arms correspond to the two educational interventions designed for this study. In the randomized group the three arms will be: intervention A, intervention B and a control arm.

The randomization will be made using the block randomization technique. The blocks consist of six patients, two subjects per group or three patients, one patient per group (Intervention A, Intervention B and control). The blocks will be marked with a number from 1 to 96 and they will be chosen at random to create the allocation sequence using a sequence of random numbers generated by the Microsoft Excel 2003 program with the function fx:RAND(). The assignment to this group will be made by contacting the person responsible for random sequence by phone. Because the patients are from various health centers, the randomization and the presence of the two interventions and control subjects in all the health centers will be guaranteed.

After randomization all the study data will be recorded and the performance of correct inhalation techniques will be measured in all groups (inclusion visit). In the case of the control group, the inhalation techniques will be tested asking them about how they use their inhalers and the interviewer will write down the mistakes in the template designed. The interviewer will only correct the critical mistakes. In the follow-up visits the subjects will be invited to perform the inhalation technique and the mistakes will be written down.

### Follow-up

All study groups will undergo the same follow up: five visits during one year

#### Control group

Visit one will take place one month after the inclusion visit. Primary and secondary outcomes will be measured (excluding spirometry and quality of life).

Visit two will take place three months after the inclusion visit. All the study data will be recorded.

Visit three will take place six months after the inclusion visit. Primary and secondary outcomes will be measured (excluding spirometry and quality of life).

Visit four will take place 12 months after the inclusion visit. All the study data will be recorded.

#### Intervention A group

Visit one will take place one month after the inclusion visit. Primary and secondary outcomes will be measured (excluding spirometry and quality of life) and correct inhalation techniques using the designed leaflet will be encouraged.

Visit two will take place three months after the inclusion visit. All the study data will be recorded and correct inhalation techniques using the designed leaflet will be encouraged.

Visit three will take place six months after the inclusion visit. Primary and secondary outcomes will be measured (excluding spirometry and quality of life) and correct inhalation techniques using the designed leaflet will be encouraged.

Visit four will take place 12 months after the inclusion visit. All the study data will be recorded and correct inhalation techniques using the designed leaflet will be encouraged.

#### Intervention B group

Visit one will take place one month after the inclusion visit. Primary and secondary outcomes will be measured (excluding spirometry and quality of life), correct inhalation techniques using the leaflet will be encouraged and training by the monitor will focus on motivational aspects.

Visit two will take place three months after the inclusion visit. All the study data will be recorded and correct inhalation techniques (using both the leaflet and the training) will be encouraged.

Visit three will take place six months after the inclusion visit. Primary and secondary outcomes will be measured (excluding spirometry and quality of life) and correct inhalation techniques using the leaflet will be encouraged and training by the monitor will focus on motivational aspects.

Visit four will take place 12 months after the inclusion visit. All the study data will be recorded, correct inhalation techniques using the leaflet will be encouraged and the training with the monitor will focus on motivational aspects.

### Statistical analysis

A descriptive statistical analysis will be performed for all the study variables. We will calculate the mean, median and standard deviations for quantitative variables, and the absolute and relative frequency for qualitative variables.

The 95 % confidence interval will be applied. The analysis will be made following an intention-to-treat procedure. The baseline comparison will be made between the main variables that we expected to be related to the primary outcome using the Chi-Square test or an analysis of variance (ANOVA).

The between-group comparison for the primary outcome will be explored using the Chi-Square Test. The Relative Risk Reduction (RRR), the Absolute Risk Reduction (ARR) and the Number Needed to Treat (NNT) will be calculated. Inferences for the secondary outcomes will be made using an ANOVA or Chi-Square test. Each group will be analyzed separately. The comparison using PPS groups is unreliable because of the presence of unknown and uncontrolled confounders. It is necessary to make a comparison between RCT groups alone and analyses which include the PPS groups should be treated as observational studies with known confounding factors adjusted for in the analysis. Then the analysis will be performed following the next steps:

Comparison in RCT group: this will be made between each intervention arm versus control arm (intervA versus control; intervB versus control) and between intervention A and intervention B.

Comparison between RCT and PPS groups: this will be performed between the intervention arms of each group (intervA RCT group versus intervA PPS group; intervB RCT group versus intervB PPS group).

Finally, a logistic regression model will be performed for the primary outcome (performance of correct inhalation technique (yes/no)), considering the intervention as the predictive variable and the rest of the independent measures as the possible modifying factors. We will use the usual 5 % significance level (α = 0.05) and the SPSS statistical package, version 15.0, to run the proposed analysis.

### Study limitations

The first limitation that we consider is due to the design of study. Designs that allow participants to choose their treatment are susceptible to confounding factors (including the preference of patients). That is, the characteristics of subjects who choose one treatment may differ from those who choose the other in ways that are related to the outcomes of interest. This weakens the internal validity of the experiment and makes it impossible to isolate the ‘pure’ effect of the respective treatments [31] although this can be controlled with the comparison of characteristics between groups. Related to external validity, almost all eligible patients enter the study, allowing examination of patient characteristics with all the strengths of preferences [32].

As patients’ preferences have a ‘therapeutic effect’ on compliance and motivation, we think they can have a positive effect on patients´ inhalation skills. These effects are similar to the placebo effect [32]. Another related aspect is the Hawthorne effect during the course of the study (that is, the tendency of subjects participating in a research study to change their behavior). Although this could affect overall estimates of the adherence, the implications might be less important in comparing results between the intervention and the control group; furthermore, it is difficult to perceive that any potential Hawthorne effect would be maintained over the many months of this study. Both effects will be taken into account during the analysis.

Another limitation is the selection bias due to missing data. In order to diminish this bias, we will apply several strategies:

An increase of 40 % in the sample size (expected losses)

Three phone calls on different days and times for unreachable patients

Rescue appointments for those who do not attend scheduled visits (three different appointments)

In addition, we take into account the possible contamination between the control and the intervention group because of the relationship between subjects in their daily life (neighborhood, relatives, social networks or associations). However, in another educational intervention study performed with obese patients in our area we did not find a significant level of this effect [33]. We also believe that the intervention characteristics (several steps and individual visits) that we have described will have little influence on contamination between groups.

Another important aspect to consider is the protocol and the intervention standardization. This is why the dynamic has been structured in an exhaustive way and the intervention will be performed by two professionals trained in communication, disease knowledge and inhalation techniques of the different devices used by COPD patients. Furthermore, we have designed a manual for the researchers where we explain the working plan, the different parts of the intervention, the protocol scheme to know what they have to measure each time and the details to assess each variable included in the study. In this way, the procedure can be replicated elsewhere.

## Discussion

We designed and developed a multifactor intervention to improve adherence in COPD, the ICEPOC study [34]. This study permitted us to analyze deeply the motives and barriers or difficulties that these patients have in complying with the recommended medication regimens. This multifactor intervention included training about inhalation techniques and we could see that up to 75 % of patients do not perform a correct inhalation technique. When the intervention was finished only 17 % did not perform a correct inhalation technique (data not published). Based on this observation, our hypothesis is that the application of two educational interventions in patients with COPD who use inhaled therapy is going to improve by at least 25 % the number of patients who perform the inhalation technique correctly.

RCT are regarded as the gold standard for assessing the effectiveness of treatments. Random allocation of patients reduces the probability that bias will affect the outcome of the trial [35]. Such designs have the power to eliminate a variety of alternative explanations for changes in health-related measures over time, permitting the researcher to reasonably conclude that the intervention itself caused the changes. Although random allocation is intended to evenly distribute characteristics of participants that may affect outcome and to remove selection bias, it may not deal with other potential biases. One of these is patients’ preferences [22]. Because patients’ preferences are not dealt with in the randomization process, they are viewed as a potential threat to the validity of trials [23]. The effect of patients’ preferences on treatment outcomes in RCTs is, however, uncertain [22].

Patients with strong preferences may decline to participate. In a trial in which strong preferences exist and a large number of patients refuse randomization, the external validity will be adversely affected. When this occurs, generalizability of the results to a wider population will be limited. If patients with preferences consent to randomization, this may also affect internal validity of the trial. In this case some patients will receive their preferred treatment and others will not. Those who receive their preferred treatment might be better motivated and comply better with the treatment programs and report better outcomes [32]. On the other hand, patients who do not receive their preferred treatment may experience ‘resentful demoralization’ and may be less motivated, less adherent to the treatment program, and even drop out of the trial. The effects of preference are likely to be more apparent in unblinded trials, such as educational interventions, in which patients are aware of the treatment they are receiving and the outcome measure is subjective and self reported by them [22]. Additionally, the opportunity to choose a treatment based on personal preferences may enhance an individual’s sense of control over the learning process within the context of an educational/behavioral program, thereby increasing self-efficacy for changes in behavior and resulting in enhanced outcomes.

One approach to dealing with patients’ preferences is the partially randomized preference design also known as a patient preference trial or comprehensive cohort design [23,32]. Patient preference design complements, but does not replace randomized trials. In this way the design of our study includes a preference group and a randomized group. We have calculated that 99 patients need to be included in each arm, both in the RCT group (three arms) and in the PPS group (two arms). As it is possible that we will find more patients with preferences by any of the interventions, we have established an upper limit in the preference group of 120 patients per arm; so, if we get this number of patients included in any preference arm we will stop the inclusion of additional patients in this group.

Measuring patient preferences within a fully randomized design deserves further use as this conserves all the advantages of a fully randomized design with the additional benefit of allowing for the interaction between preference and outcome to be assessed [36]. With the trend toward active patient participation in health care decisions, it is likely that such preferences may be a key factor in determining the effectiveness of health education programs. The preference trial is an alternative to the RCT because it is more similar to decision-making in a clinical setting [31].

### Trial status

The TECEPOC study is in an activating recruiting phase since 2010.

This study has been approved by the Ethical Committees of Distrito Sanitario Málaga (07/07/2009) and Axarquía (16/02/2010) and by Committee of Clinical Trials of Comunidad Autónoma de Andalucía (25/01/2011). Subjects will give their informed consent before being enrolled in the study. Study completion date is estimated to be January 2013. Current Controlled Trials ISRTCTN15106246

## Competing interests

The authors declare that they have no competing interests.

## Authors´ contributions

PBF has been involved in drafting the manuscript and writing it. She has participated in the design of the study and the intervention. JLF has been involved in the design of the study and he has participated in reviewing the manuscript. FZS has been involved in the design of the intervention and he has participated in reviewing the manuscript. AGR has been involved in the design of the study and he has participated in reviewing the manuscript. DPT has been involved in the design of the study, and he has participated in reviewing the manuscript. FLF has been involved in drafting the manuscript and writing it. She has participated in the design of the study and the intervention. All authors read and approved the final manuscript.
